# Current trends in site-specific lymph node dissection for upper tract urothelial carcinoma: precise staging and survival

**DOI:** 10.1097/JS9.0000000000002129

**Published:** 2024-10-22

**Authors:** Yige Jia, Kan Wu, Xiang Li

**Affiliations:** Department of Urology, Institute of Urology, West China Hospital, Sichuan University, Chengdu, People’s Republic of China

*Dear Editor,*

The POUT trial has established the importance of adjuvant chemotherapy following radical nephroureterectomy (RNU) for patients with upper tract urothelial carcinoma (UTUC), encompassing individuals with muscle-invasive disease or positive lymph node status^1^. Since it is hard to predict whether a patient has muscle-invasive disease before surgery, the necessity of performing lymph node dissection (LND) to assist staging becomes an important issue. Moreover, the optimal template of LND in RNU remains controversial. Our objective was to evaluate the utilization situation of LND, and enhance the existing template for precise staging and treatment, with a particular focus on site-specific characteristics, utilizing the Surveillance, Epidemiology, and End Results (SEER) database.

A total of 7366 patients with histologically confirmed UTUC who underwent RNU were identified from the SEER database. The rate of LND utilization exhibited an upward trend over the past two decades, with the rate being higher for the left renal pelvis than the right renal pelvis, while the trend of LND in the ureter appeared more erratic (Fig. 1A). The distribution of estimated lymph nodes (ELNs) in different sites exhibited a comparable pattern, with the highest proportion of patients having only one lymph node removed, decreasing gradually as the ELN count increased (Fig. 1B). Notably, as illustrated in Figure 1C, the proportion of nodal status at different sites post-LND displays a significant difference. There were notably fewer cases of N+ ureteral cancers diagnosed, and the incidence of N+ disease was similar between the left and right renal pelvis, despite a higher number of patients undergoing LND.

**Figure 1:**
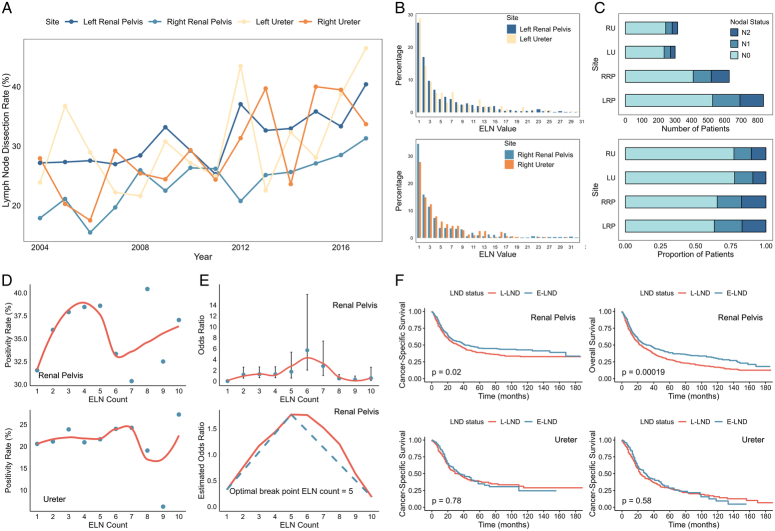
Trends in site-specific lymph node dissection (LND) for upper tract urothelial carcinoma (UTUC). (A) Temporal trends in the rate of LND during nephroureterectomy across different sites. (B) Distribution of the number of harvested lymph nodes at different sites. ELN, examined lymph nodes. (C) Number and proportion of patients by different nodal status after LND at different sites. LU, left ureter; LRP, left renal pelvis; RU, right ureter; RRP, right renal pelvis. (D) Scatterplot showing the relationship between ELN counts and the proportion of patients with positive nodes. (E) LOWESS smoother curves and Chow test showing the association between odds ratio (negative-to-positive nodes) and the number of examined lymph nodes. LOWESS, Locally Weighted Scatterplot Smoothing. (F) Kaplan–Meier survival curves for cancer-specific survival and overall survival comparing different extents of LND. E-LND, extended LND; L-LND, localized LND.

Furthermore, we examined the optimal number of LNDs in the renal pelvis and ureter. With an increase in ELN count, the rate of N+ disease rose within a specific range for the renal pelvis but remained relatively stable for the ureter (Fig. 1D). Based on the hypothesis that a higher ELN count offers a better chance of identifying positive LNs, we utilized binary logistic regression to evaluate the odds ratios (ORs, node-negative versus node-positive) for each ELN count, fitting them with Locally Weighted Scatterplot Smoothing (LOWESS) using a bandwidth of 2/3 to determine the optimal number for precise staging, utilizing the Chow test to find structural breakpoints. We suggest five ELNs to be an optimal choice for LND in the renal pelvis (Fig. 1E), but the number of ELNs for staging in the ureter requires further discussion. Previous studies have demonstrated the positive impact of LND, particularly extended LND involving more than four LNs in T3/4 patients^2^. Consequently, we constructed Kaplan–Meier curves to compare the prognostic value between extended LND and localized LND. As depicted in Figure 1F, extended LND improves both overall survival (OS) and cancer-specific survival (CSS) in the renal pelvis but does not improve survival outcomes in the context of the ureter.

Researchers have summarized the role of LND into two main categories: staging and therapeutic roles. Given the variability in lymphatic drainage within the upper urinary tract, it is essential to take site-specific factors into account during these surgeries^3^. There are several interesting findings in our study. Firstly, with a similar rate of LND and ELN count, ureteral cancer showed a lower positivity rate. Current research indicates that individuals with tumors in the renal pelvis and upper ureter should undergo a template dissection involving the hilum, paracaval, and paraaortic nodes, those with lower ureteral tumors should undergo iliac lymph node dissection^4^. Thus, the issue of whether there is a decreased metastasis rate or a less standard LND in ureteral tumors requires further examination. Secondly, a greater number of ELNs correlated with enhanced staging accuracy and better OS and CSS in the renal pelvis. Most patients have only one lymph node examined, reflecting the lack of consensus on LND. Previous studies have recommended dissecting six to eight lymph nodes for UTUC patients to improve staging accuracy and prognosis^3^. In our study, an extended LND involving at least five lymph nodes is recommended for patients with renal pelvis cancer. Furthermore, lymph node dissection for ureteral tumors requires careful consideration. Increasing the number of dissections did not seem to improve the detection of N+ diseases, or enhance survival. Similar results were found in a prospective study, where patients with renal pelvic tumors had significantly better survival following template-based LND, but no such benefit was observed for those with ureteral involvement^5^.

In conclusion, these findings highlight the demand for higher-quality templates for LND in UTUC and more standardized treatment protocols, which will lead to improvements in subsequent adjuvant therapy and patient survival.

## Ethical approval

This study did not require further ethics committee approval as it is a retrospective study based on a public database.

## Consent

This study is a retrospective study based on a public database and does not involve the privacy of patients, so it was conducted without informed written consent.

## Source of funding

None.

## Author contribution

Y.J.: writing – original draft preparation, conceptualization, methodology, and software; K.W.: data curation, visualization, and investigation; K.W. and X.L.: supervision, writing – reviewing, and editing.

## Conflicts of interest disclosure

The authors declare no conflicts of interest.

## Research registration unique identifying number (UIN)

Clinical trial registration: none.

## Guarantor

All authors.

## Data availability statement

The data that support the findings of this study are openly available in seer.cancer.gov.

## Provenance and peer review

Not invited.
